# Organic Porous Materials and Their Nanohybrids for Next-Generation Thermoelectric Application

**DOI:** 10.1021/acsami.4c12729

**Published:** 2024-11-22

**Authors:** Meng-Hao Lin, Shao-Huan Hong, Jian-Fa Ding, Cheng-Liang Liu

**Affiliations:** † Department of Materials Science and Engineering, National Taiwan University, Taipei 10617, Taiwan; ‡ Institute of Polymer Science and Engineering, National Taiwan University, Taipei 10617, Taiwan; § Advanced Research Center for Green Materials Science and Technology, National Taiwan University, Taipei 10617, Taiwan

**Keywords:** porous materials, conducting fillers, thermoelectric, organic hybrids, low thermal conductivity

## Abstract

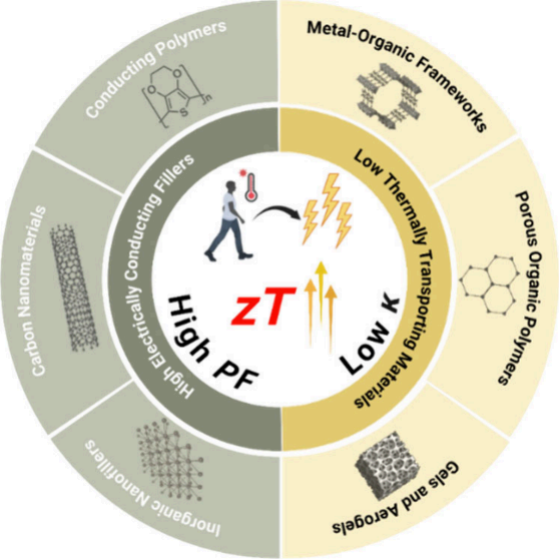

Thermoelectricity offers a promising solution for reducing carbon emissions by efficiently converting waste heat into electrical energy. However, high-performance thermoelectric materials predominantly consist of rare, toxic, and costly inorganic compounds. Therefore, the development of alternating material systems for high-performance thermoelectric materials is crucial for broader applications. A significant challenge in this field is the strong interdependence of the various thermoelectric parameters, which complicates their simultaneous optimization. Consequently, the methods for decoupling these parameters are required. In this respect, composite technology has emerged as an effective strategy that leverages the advantages of diverse components to enhance the overall performance. After elaborating on the fundamental concepts of thermoelectricity and the challenges in enhancing the thermoelectric performance, the present review provides a comparative analysis of inorganic and organic materials and explores various methods for decoupling the thermoelectric parameters. In addition, the benefits of composite systems are emphasized and a range of low thermal conductivity materials with microporous to macroporous structures are introduced, highlighting their potential thermoelectric applications. Furthermore, the current development obstacles are discussed, and several cutting-edge studies are highlighted, with a focus on the role of high electrical conductivity fillers in enhancing the performance and mechanical properties. Finally, by combining low thermal conductivity materials with high electrical conductivity fillers can achieve superior thermoelectric performance. These insights are intended to guide future research and development in the field of organic porous materials and their nanohybrids in order to promote more sustainable and efficient energy solutions.

## 1 Introduction

The contemporary energy crisis and environmental degradation, along with associated geopolitical tensions, present a complex and unavoidable challenge that necessitates dedicated research effort. Currently, energy generation predominantly relies on the combustion of fossil fuel in a heat engine, followed by conversion of the resultant mechanical energy into electricity. Specifically, global electricity generation relies heavily on fossil fuels (∼67%), hydropower (16%), and nuclear energy (11%), with modest but escalating contributions from wind (>4%) and solar energy (>2%).^1^ However, the conversion of energy from primary source to electricity is often accompanied by significant loss in the form of waste heat, with typical conversion efficiencies ranging from 35–50% for heat engines, 20% for solar thermal facilities, and 15–40% for solar cells.^1^ Furthermore, over 50% of the available natural and waste heat exists as warm fluids (*T* < 250 °C), and no viable technology can currently harness electricity from this low energy-density heat.^2−5^

With a view to achieving the goal of net-zero carbon emissions by 2050, as proposed by the 26th UN Climate Change Conference of the Parties (COP26) in 2021, there has been increased focus on advancing renewable, carbon-neutral energy replacements. In this context, thermoelectricity has emerged as a compelling candidate for addressing the energy crisis from an environmentally sustainable perspective.^6,7^ Thermoelectric materials possess the remarkable ability to capture waste heat directly and convert it into electricity via the thermoelectric effect, offering advantages such as low maintenance requirements, independence from the type of heat source, and simplified setup.^8^ Consequently, thermoelectricity provides a viable means of harnessing waste heat from diverse sources, including solar radiation, geothermal energy, and various human activities. Moreover, the generation of thermoelectricity requires only the presence of a temperature gradient, thus enabling it to provide a continuous power supply over the long-term, regardless of weather conditions, location, or time constraints.^9−11^

In this review, the fundamental concept of thermoelectricity and the challenges associated with enhancing its performance are first elaborated. Then, inorganic and organic materials are compared, along with several methods for decoupling the various thermoelectric parameters, and the advantages of composite systems for thermoelectric applications are discussed. Additionally, various classes of low thermal conductivity materials with microporous to macroporous structures are introduced, and the current obstacles in their development for practical application in composite thermoelectric systems are discussed. Furthermore, several cutting-edge studies based on these concepts and their relevant performance metrics are compared. Various types of high electrical conductivity fillers are introduced, and their potential to improve the overall thermoelectric performance is discussed, along with their ability to enhance the mechanical properties via their specific nanostructures. Lastly, the review provides a summary and outlook for future thermoelectric development. The aim is to present a novel concept for the development of high-performance thermoelectric composite systems by combining low thermal conductivity materials with high electrical conductivity fillers to create a phonon glass electron crystal scenario.

## 2 Basic Concept of Thermoelectricity

Thermoelectricity can be understood through two fundamental effects: the Seebeck effect and the Peltier effect. This section will briefly introduce the working principles and relevant factors of these effects. Additionally, a direct comparison between organic-based and inorganic-based thermoelectric materials is provided to offer a comprehensive overview.

### 2.1 Thermoelectric Effect

**Figure 1:**
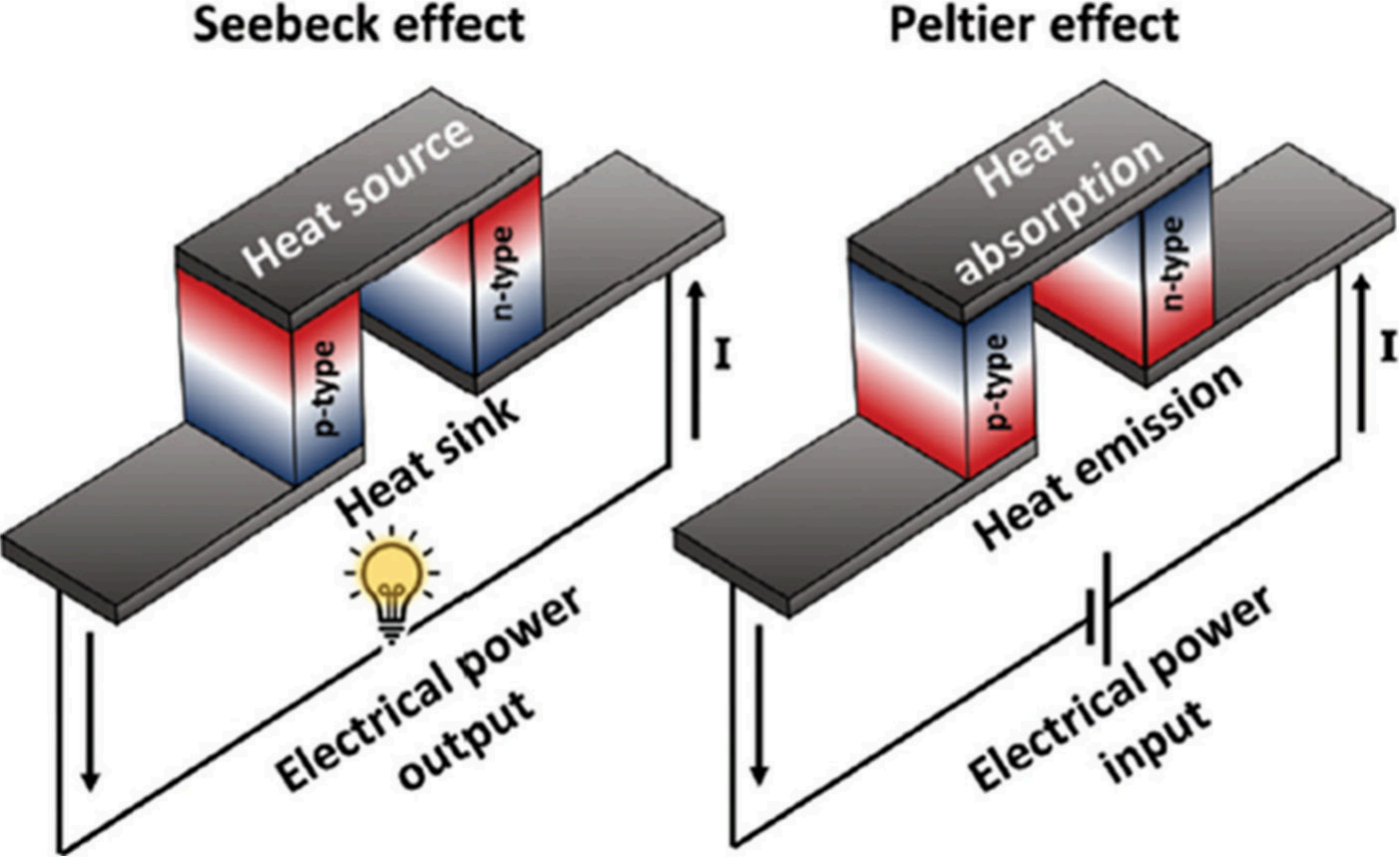
Illustration of Seebeck effect and Peltier effect. Reproduced from with permission from ref (14). Copyright 2022, Royal Society of Chemistry.

The thermoelectric effect is defined as a phenomenon wherein a temperature gradient within a semiconductor or conductor induces the creation of an inherent electrical field.^12^ In the forward thermoelectric effect, first observed by T. J. Seebeck in 1821,^12^ the higher energy levels of electrons or holes at the hot end causes them to migrate toward the cold end to establish a temperature equilibrium, assuming that no external heat source maintains a constant thermal gradient. This directional flow of charge carriers leads to an accumulation of more negative or positive charges at the cold end, thus resulting in the generation of an electromotive force or thermovoltage described by eq 1:
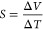
1where *S* is the Seebeck coefficient, Δ*V* is the voltage gradient, and Δ*T* is the temperature gradient. Conversely, in the reverse thermoelectric effect, known as the Peltier effect, was discovered by J. C. Peltier in 1834.^13^ In this scenario, an external electromotive force is applied to a circuit containing two junctions composed of different conductors, such that one junction experiences heating while the other undergoes cooling in order to equalize the potential difference between the two conductors. As the charge carriers are transported through the distinct conductors, the energy difference is offset by energy exchange between the two junctions. During this process, heat is either absorbed or dissipated depending on the direction of the electric current flow, as shown in Figure 1.^14^

The energy conversion effectiveness of a thermoelectric material is usually evaluated by using the dimensionless figure of merit (*zT*), which is defined by eq 2:
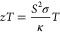
2where σ and κ are the electrical and thermal conductivity, respectively, and *T* is the working temperature. Note that the factor *S*^2^σ in eq 2 is also known as the power factor (*PF*). Once the *zT* value is obtained, the maximum heat-to-power conversion efficiency (η_max_) can be estimated by using eq 3:
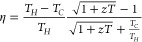
3where *T*_H_ and *T*_C_ are the respective temperatures of the hot and cold reservoirs.^15^ An examination of the above equations indicates that an enhanced *zT* value can directly contribute to an increased energy conversion efficiency. However, the primary challenge in further improving *zT* arises from the strong coupling of the thermoelectric parameters σ, *S*, and κ. Various strategies for decoupling these parameters include: (i) improving the electrical conductivity/Seebeck coefficient by implementing quantum confinement in one or more dimensions,^16,17^ (ii) mitigating the phononic contribution of thermal conductivity through scattering mechanisms at grain boundaries, interfaces, pores, and defects while preserving the electronic properties,^18,19^ (iii) implementing energy filtering at introduced barriers (e.g., interfaces) and employing structural and electronic modulations to modulate energy transitions via band structure engineering,^20^ and (iv) creating a thermoelectric composite by combining several material systems and utilizing their respective benefits.^21−23^

### 2.2 Inorganic vs. Organic Thermoelectric Materials

Since the advent of thermoelectric materials, inorganic compounds such as bismuth telluride (Bi_2_Te_3_) and its alloys, lead telluride (PbTe) and its alloys, silicon–germanium (SiGe) alloys, antimony telluride (Sb_2_Te_3_), and tin selenide (SnSe) have been extensively investigated due to their potentially high *zT* values.^10,24−26^ However, their widespread application is impeded by several factors. First, the scarcity of specific elements, notably tellurium (Te), poses a significant challenge. The abundance of Te in the Earth’s crust is only around 0.001 ppm, which is even lower than that of gold (Au) at 0.004 ppm.^27^ Moreover, the high working temperature requirements, toxicity, and brittleness further limit the applicability of these materials in various scenarios. Furthermore, because inorganic thermoelectric compounds are always identified as electron crystals, their *zT* optimization methods are focused on minimizing their intrinsically high thermal conductivities.^28−31^

Meanwhile, significant strides have recently been made in the development of organic thermoelectric materials, which offer several advantages such as low weight, mechanical flexibility, affordability, nontoxicity, abundance of raw materials, solution processing over large areas, and intrinsically low thermal conductivity.^32−36^ Consequently, organic thermoelectric materials are viewed as having high potential to complement inorganic thermoelectric compounds. However, owing to the inferior electrical properties of organic materials, their *PF* values tend to be much lower than those of inorganic compounds. Nevertheless, organic thermoelectric materials are typically characterized as phonon glasses, which have intrinsically low thermal conductivity. Therefore, strategies aimed at enhancing the *zT* value of organic thermoelectric materials are primarily focused on increasing their *PF* values.^37−40^

## 3 The Development of Composite Thermoelectric Materials

Considering the intrinsically low thermal conductivity of organic thermoelectric materials and the relatively high electrical conductivity of inorganic thermoelectric compounds, the synergistic optimization of both properties can be pursued via the preparation of inorganic/organic hybrids that leverage the inherent advantages of each component.^41−44^ Compared to single-component thermoelectric materials, thermoelectric hybrids hold the potential to achieve a higher thermoelectric performance than that of each individual component. Furthermore, beyond the individual effects of the components in thermoelectric hybrids, the interfacial properties between organic and inorganic fillers play a crucial role in determining the thermoelectric performance. For example, in the so-called phonon block-electron tunnel hybrids, the phonons and electrons are scattered differently at the interfaces such that electrons can quantum tunnel through the interface while a substantial thermal impedance mismatch exists between the two materials. This “selective interface” effect can lead to thermoelectric hybrids with simultaneously high electrical conductivity and low thermal conductivity.^45−49^ Besides, the interface between inorganic and organic materials could form an energy filtering effect, which high-energy carriers could jump through the interface while the low-energy carriers are restricted in components. This effect could enhance the Seebeck coefficient. Some strategies could control the interface between inorganic/organic hybrids, such as establishing the organic–inorganic superlattice structure, introducing low-dimensional materials, and desiring a suitable organic structure to establish the interaction between inorganic and organic components.^50,51^

### 3.1 Low Thermal Transporting Materials

The above discussion suggests that the use of a low thermal conductivity material as the filler of a thermoelectric composite might be a promising approach to achieving a high *zT* value.^52^ Therefore, an understanding of methods for controlling the thermal conductivity becomes crucial. The thermal energy transport is primarily determined by both the electronic contribution (κ_e_) and the lattice contribution (κ_l_). For the former, the Wiedemann–Franz law states that the ratio between the electronic contribution and the electrical conductivity is proportional to the temperature, while the latter originates from lattice vibrations.^53,54^ It follows that reducing either κ_e_ or κ_l_ can result in a decreased thermal conductivity. However, reducing κ_e_ also leads to a decrease in the electrical conductivity, which conflicts with the goal of *zT* enhancement. Therefore, reducing κ_l_ is considered a favorable strategy for lowering the thermal conductivity.^55,56^

**Figure 2:**
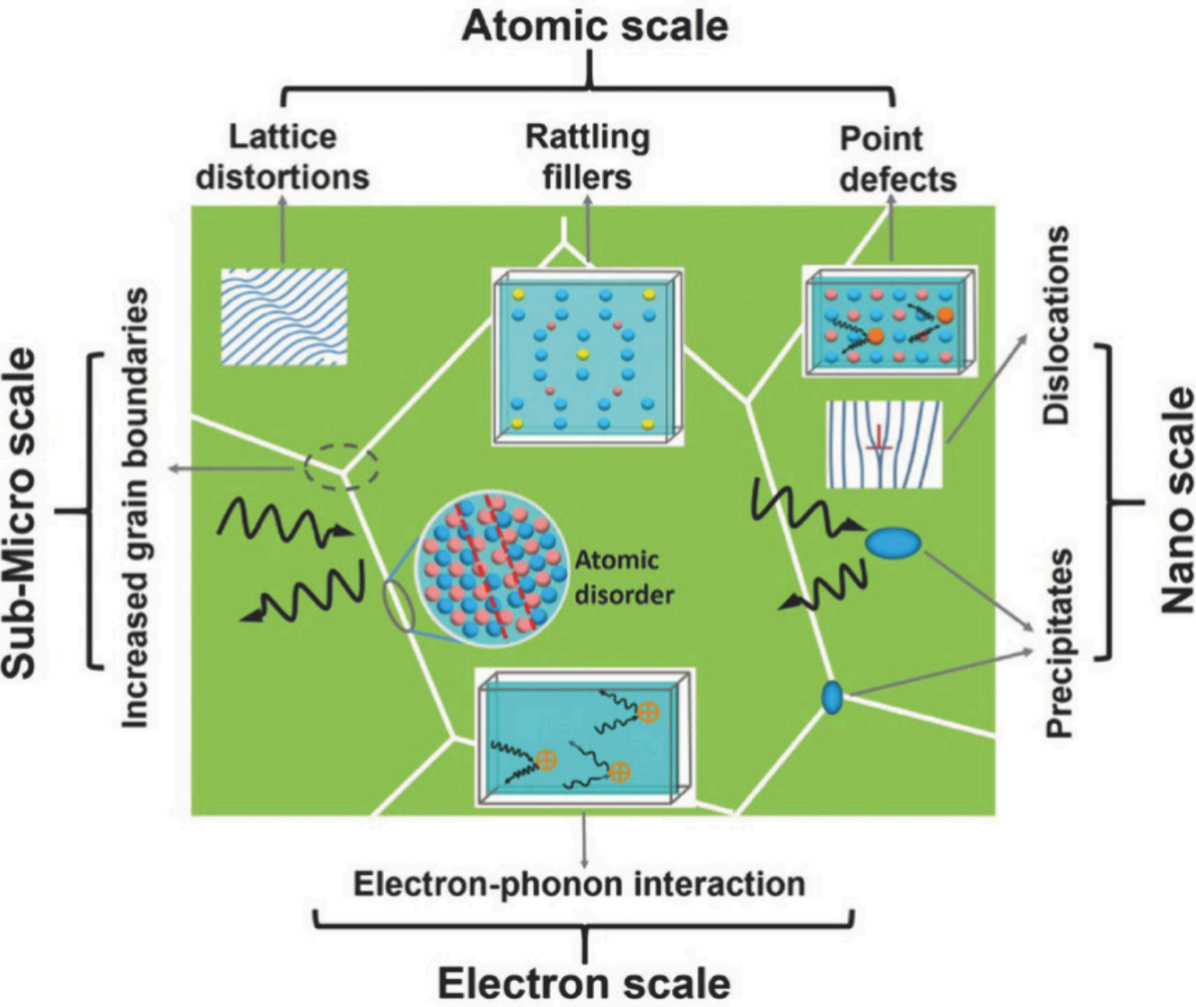
Typical phonon scattering effect in thermoelectric materials to reduce thermal conductivity. Reproduced from with permission from ref (59). Copyright 2017, Wiley-VCH.

Recently, the introduction of porosity into materials has emerged as a favorable method for decreasing the thermal conductivity due to strong phonon scattering,^57,58^ as illustrated in Figure 2.^59^

For effective reduction of the thermal conductivity, the pore size of the material should ideally be smaller than the mean free path of air molecules (∼70 nm). Additionally, lowering the material density is crucial for achieving a low thermal conductivity. Porosity serves a dual role in this context by lowering the thermal conductivity and providing numerous host sites for dopant molecules, thereby enhancing the charge carrier density. Hence, several organic porous materials with potential use in the development of thermoelectric composites are introduced in the following three subsections, their relevant thermal conductivity properties are also listed in Table 1.

**Table 1:** Thermal Conductivity Value of Numerous Porous-Based Material Systems

Compound	Application	κ [W m^–1^ K^–1^]	Test method	Reference
Ni_3_(HITP)_2_	thermoelectricity	0.21	home-built steady-state	(60)
Ni_3_(HITP)_2_@CNT p-type	thermoelectricity	0.82	laser flash	(61)
Ni_3_(HITP)_2_@CNT n-type	thermoelectricity	0.64	laser flash	(61)
Ni-PTC	thermoelectricity	0.20	modified transient plane source	(62)
Cu_3_(BTC)_2_@TCNQ	thermoelectricity	0.25	TDTR	(63)
Cu-BHT	thermoelectricity	1.75	home-built thermal conduction	(64)
20-Zr-MOF/PAn/PSS film	thermoelectricity	0.46	ASTM D5470	(65)
CPP-15 Pellet	thermoelectricity	0.021	laser flash	(19)
PANi@MOF-801	thermoelectricity	0.023	transient plane source	(66)
MOF-5	N/A	0.32	longitudinal, steady-state heat flow	(67)
Zr-DPA/EP	energy management	0.16	laser flash	(68)
NF@Co/C-550	thermal insulation	0.51	laser flash	(69)
CNF@Al-MIL-53 aerogel	thermal insulation	0.041	transient plane source	(70)
COF-300	N/A	0.048	modified transient plane source	(71)
RIO-1	N/A	0.039	modified transient plane source	(71)
RIO-4	N/A	0.043	modified transient plane source	(71)
RIO-20	N/A	0.039	modified transient plane source	(71)
HAP_2_–NCMP aerogel	thermal insulation	0.025	transient plane source	(72)
CMP-ED aerogel	thermal insulation	0.034	transient plane source	(73)
PVA/PEDOT:PSS/Te-NWs hydrogel	thermoelectricity	0.468	Transient hot wire	(74)
pAMPS/pSBAA hydrogel	thermoelectricity	0.50	transient plane source	(75)
DN5C–P hydrogel	thermoelectricity	0.68	transient plane source	(76)
MCNT–TiO_2_–SiO_2_–TiN hydrogel	solar steam evaporation	0.72	heat flux calculation	(77)
cement-PVA hydrogel composite	thermal insulation	0.21	transient plane source	(78)
PNIPAm hydrogel	smart window	0.47	ASTM D5470	(79)
SiZrOC aerogel	thermal insulation	0.03	transient plane source	(80)
N-doped graphene aerogel	thermal insulation	0.02	transient plane source	(81)
PEDOT-Tos/SWCNTs aerogel	thermoelectricity	0.09–0.24	3ω method	(82)
PEDOT:PSS/SWCNT aerogel	thermoelectricity	0.074	transient plane source	(83)

#### 3.1.1 Metal–Organic Frameworks

Metal–organic frameworks (MOFs), characterized by porous coordination polymers composed of inorganic metal nodes and organic linkers, have emerged as highly promising and versatile materials.^84,85^ Their inherently high porosity and tunable physical and chemical properties render them exceptionally well suited for a wide range of applications, particularly in the realm of thermoelectric devices.^86^ By meticulously selecting and arranging metal centers and organic ligands to modulate their structural topologies, the thermoelectric properties of the MOFs can be easily tailored.^87^

**Figure 3:**
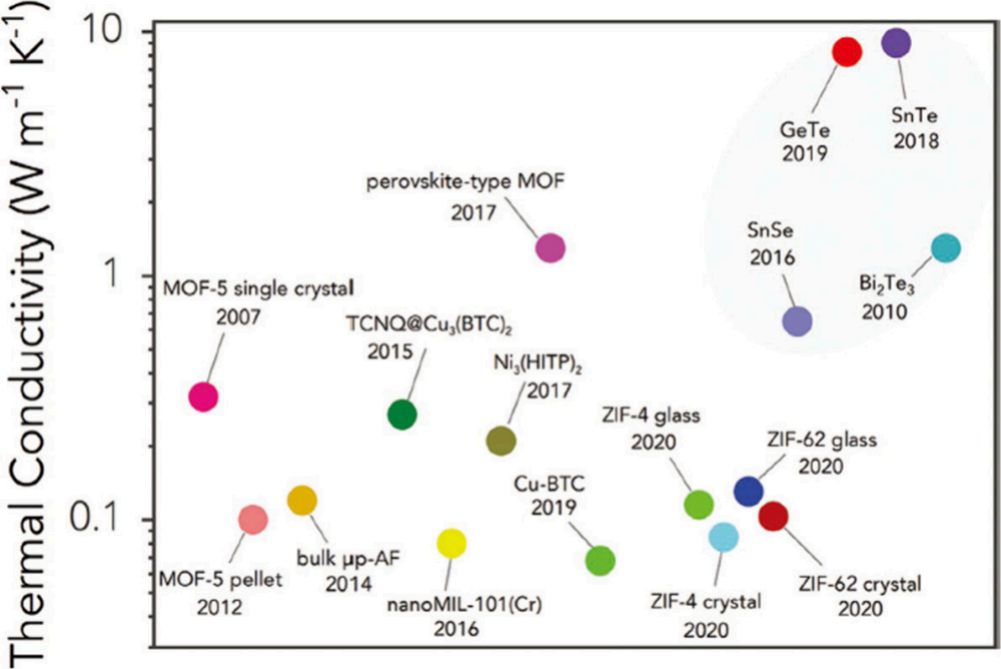
Experimental data of thermal conductivity measured at room temperature for various MOFs compared to some typical inorganic materials (shadowed area). Reproduced from with permission from ref (89). Copyright 2021, Wiley-VCH.

Of particular interest is the remarkable porosity of the MOF, which presents a novel avenue for enhancing the thermoelectric performance. The presence of pores facilitates strong phonon scattering, thereby reducing the thermal conductivity and concurrently enhancing the *zT* value. Recent investigations have revealed that MOFs consistently exhibit thermal conductivities below 0.4 W m^–1^ K^–1^, regardless of their structural, compositional, or morphological variations^88,89^ (Figure 3).^89^ Besides, the introduction of various substances into the pores can further influence thermal conductivity, although the underlying mechanisms remain unclear. Many current studies exploring these effects rely on theoretical calculations.^90,91^ Additionally, the porous MOF framework can serve as a space for incorporating a diverse range of guest molecules, thus leading to the development of nanohybrid systems and opening up new avenues for enhancing energy conversion efficiency.^63^ Nevertheless, the thermoelectric properties of the MOFs have yet to be extensively explored, primarily due to the challenge of achieving sufficiently high electrical conductivity. Typically, the interconnected rigid metal ions and redox-inactive organic ligands within the MOF structures create energy barriers for electron transfer, rendering them electrically insulating in nature.

**Figure 4:**
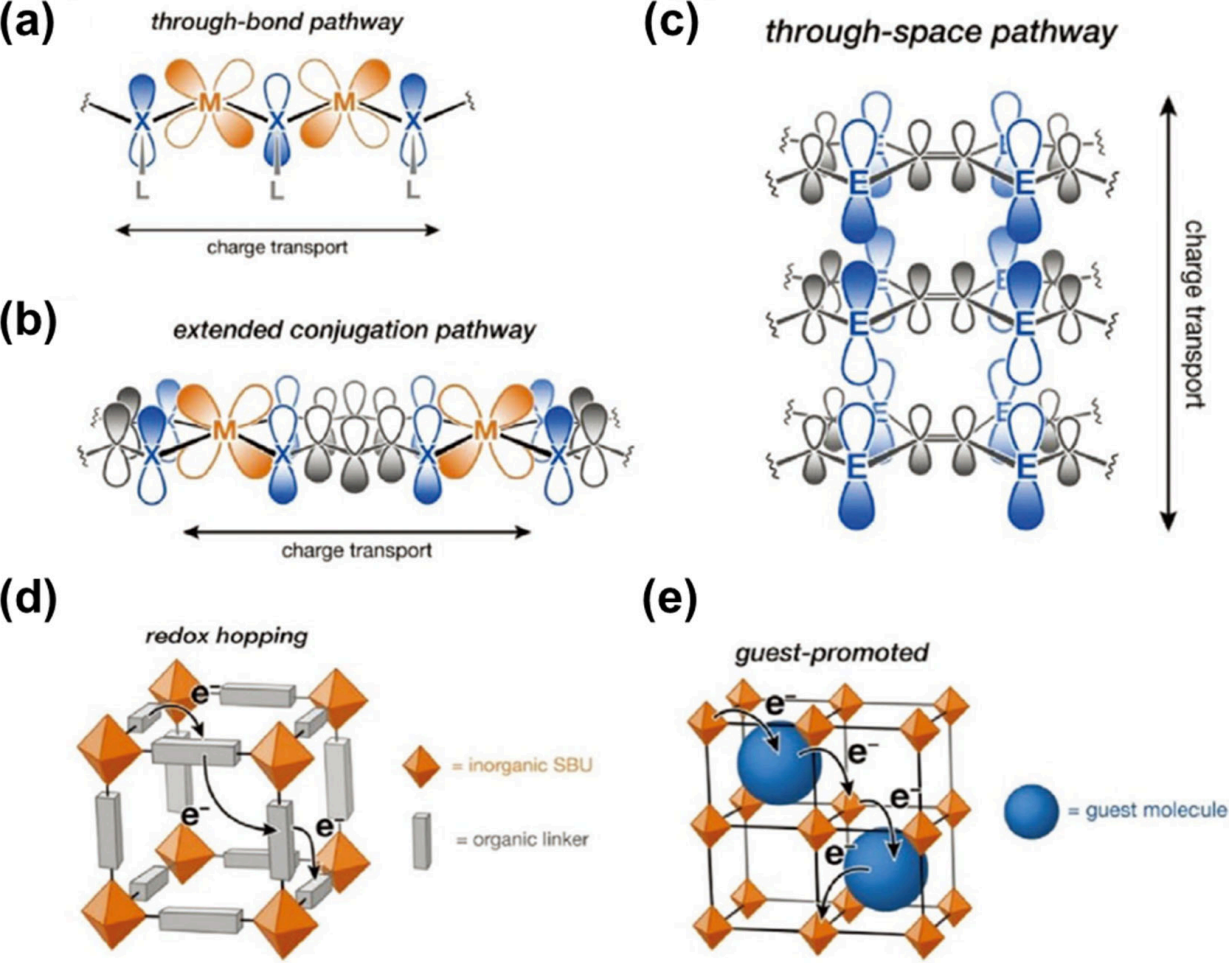
Schematic illustrations of carrier transport pathway in MOFs. (a) The through-bond pathway. (b) The extended conjugation pathway. (c) The through-space pathway. (d) Redox hopping transport. (e) Guest-promoted transport. Reproduced from ref (93). Copyright 2020, American Chemical Society.

Recently, the emergence of conductive MOFs has introduced band transport and hopping transport as viable mechanisms for enhancing their electrical characteristics,^92^ as illustrated in Figure 4.^93^ In band transport, strong interactions between electron sites facilitate the formation of continuous energy bands with delocalized charge carriers. These carriers can freely navigate through the continuous coordination or covalent bonds within the MOFs. Thus, band transport relies on the spatial and energetic overlap between orbitals of covalently linked metal ions and organic ligands.

Conversely, hopping transport involves the navigation of charge carriers between discrete energy levels, which often occurs via ligand-to-ligand, node-to-node, or ligand-to-node hopping between donors and acceptors within the MOF structure. Studies have indicated that softer and more electropositive linkers that feature sulfur or nitrogen coordination are conducive to improved energy matching between the metal and ligand orbitals. Moreover, planar MOFs with two-dimensional (2D) graphene-like structures feature extended 2D π-conjugation, which places them among the most conductive frameworks. This is because the expanded *π–d* conjugation pathways of these 2D MOFs confine the charge carriers within the 2D lattice and promote efficient charge transport along the planar direction. Moreover, hopping transport facilitates charge transfer via noncovalent *π–π* interactions, which also generates through-space pathways.^93−95^ The remarkable electrical transport achieved in these intralayer conjugation systems underscores the potential of intrinsically 2D conductive MOFs to serve as a new generation of thermoelectric materials. Consequently, a novel class of 2D semiconducting MOFs has emerged through the combination of highly conjugated organic ligands, including hexaaminobenzene, 2,3,6,7,10,11-hexahydrotriphenylene, and 2,3,6,7,10,11-hexaiminotriphenylene, with transition metal ions such as Fe, Co, Ni, Cu, and Zn.^96−99^ For instance, Dincă et al. demonstrated the fabrication of diverse semiconducting molecular frameworks with tailored electrical properties by manipulating the choice of metal ions. Notably, Ni_3_(2,3,6,7,10,11-hexaiminotriphenylene)_2_ (Ni_3_(HITP)_2_) exhibited a remarkable electrical conductivity of 58.8 S cm^–1^, a Seebeck coefficient of up to −11.9 μV K^–1^, and an ultralow thermal conductivity of 0.21 W m^–1^ K^–1^, giving *zT* values of up to 1.19 × 10^–3^.^60^ Furthermore, the authors conducted comparative analyses involving dual metal center systems, and constructed a comprehensive graph to elucidate the nuanced variations in electrical conductivity across various compositions. Their findings underscored the pivotal role of synthetic advancements in unraveling the intricate relationship between structure and electrical properties, which are crucial for advancing material development in this domain. However, there remains a pressing need for further innovations in the design of novel π-conjugated ligands and streamlined synthetic methodologies in order to realize high-performance, scalable thermoelectric materials for practical applications.

In contrast to the intrinsically conductive MOFs, where electrical transport relies on tailored combinations of metal ions and organic ligands to promote metal–ligand charge transfer or facilitate electron transfer between adjacent conjugated units, the integration of redox-active or inherently conductive guest molecules within the MOFs offers an alternative strategy for creating effective charge transport pathways.^65^ By bridging adjacent metal centers, these guest molecules establish conductive pathways that traverse the MOF structure, thereby enhancing the electrical conductivity. Moreover, MOFs with incorporated guest molecules present numerous synthetic and structural avenues for optimizing their electrical characteristics. The introduction of metal clusters, conductive polymers, or redox-active organic molecules induces conductive pathways within the material through guest–guest or guest–framework interactions.^100,101^ Additionally, the crystallinity of the guest molecules can be enhanced upon their incorporation into MOF voids, thereby promoting increased charge mobility throughout the structure. This strategy holds promise for enhancing the thermoelectric performance by improving the charge transport properties within the MOF-based composites, thus highlighting the potential of guest molecule incorporation as a versatile approach for optimizing the electrical behavior of these materials. For example, Talin et al. spearheaded groundbreaking research by infiltrating Cu_3_(benzene-1,3,5-tricarboxylate)_2_ with the redox-active reagent 7,7,8,8-tetracyanoquinodimethane (TCNQ) to achieve a notable enhancement in electrical conductivity along with a *zT* value of 7 × 10^–5^ for the TCNQ@Cu_3_(BTC)_2_ system.^63^ Additionally, the incorporation of MOFs into composites with conducting polymers or carbon-based materials has proven to be an effective strategy. For instance, Hou et al. demonstrated the effective of postsynthetic modification with polyaniline/Co-MOF-Br to obtain a superior electrical conductivity of 5 × 10^–2^ S cm^–1^ and a Seebeck coefficient of 37 μV K^–1^.^102^ This underscores the potential of conducting polymer/MOF hybrids for thermoelectric applications. Among carbon-based materials, carbon nanotubes (CNTs) stand out for their efficient charge transport and favorable mechanical properties, making them suitable candidates for integration into MOF hybrids.^103^ This integration enhances the processability and enables the creation of versatile device architectures beyond conventional pellet types. Moreover, while the high thermal conductivity of CNTs can be a limitation in certain contexts, this can be mitigated by the low thermal conductivity of the porous MOFs. For instance, Chen et al. developed a ternary flexible thermoelectric poly(3,4-ethylenedioxythiophene):polystyrenesulfonate (PEDOT:PSS)/CNTs/M-UIO-66 composite film with enhanced formability and electrical conductivity due to the incorporated CNTs.^104^ Furthermore, the same research group proposed a novel approach for the in situ crystallization of conducting MOFs on CNT surfaces, resulting in p-type thermoelectric hybrids with a record-high *zT* value of 0.04 for the as-crystallized Ni-THT/CNT composite film.^105^

#### 3.1.2 Porous Organic Polymers

**Figure 5:**
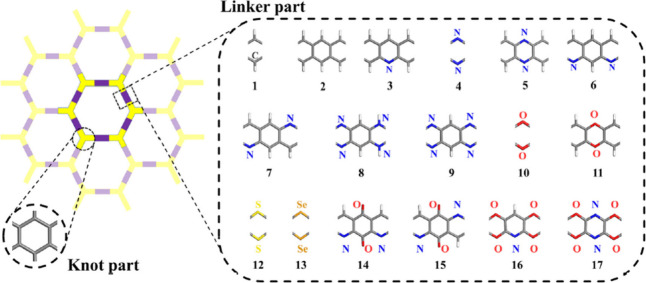
Potential chemical structures of two-dimensional COFs for thermoelectric application. The carbon, nitrogen, oxygen, sulfur, selenium, and hydrogen atoms are represented in gray, blue, red, yellow, orange, and white, respectively. Reproduced from with permission from ref (112). Copyright 2023, Royal Society of Chemistry.

Porous organic polymers (POPs) represent a burgeoning class of sustainable materials that leverage natural, abundant, and renewable precursors. Characterized by covalent bonds, the POPs have garnered significant attention as next-generation functional materials.^106^ Their intrinsic attributes, including straightforward synthetic pathways, precise control over porosity, and capacity for predesigned structure and functionality, render them highly versatile across a range of applications, including gas uptake and separation, energy and environmental technologies, organic photovoltaics, catalysis, etc. Moreover, distinct POP variants such as hyper-cross-linked polymers (HCPs), covalent organic frameworks (COFs), and conjugated microporous polymers (CMPs) have emerged, each tailored to specific applications.^107−109^ As with the MOFs, POPs such as COFs exhibit low thermal conductivity due to their porous nature. However, research into POP-based thermoelectric materials remains scarce due to their inherently low electrical conductivity and poor processability. Nevertheless, the COFs stand out as a unique type of porous crystalline material synthesized by integrating organic subunits into predictable structures following the principles of reticular chemistry.^110^ These materials consist of light elements such as B, C, N, O, and Si linked by covalent bonds, thus resulting in low density, exceptional surface area, and uniform pore size distribution.^111^ Furthermore, the predesigned skeletons and properties of COFs can be fine-tuned by judiciously selecting building units and reaction conditions to facilitate framework crystallization. In particular, 2D COFs tend to adopt nearly eclipsed stacked structures of aromatic subunits, offering oriented columnar alignments that are ideal for transporting excitons or charge carriers throughout the framework (Figure 5).^112^ Moreover, π-electron-rich or π-deficient moieties can be engineered into well-defined 2D COFs to provide next-generation electroactive organic materials.^113^ The incorporation of functional moieties such as porphyrin, thiophene, tetrathiafulvalene, and benzothiadiazole into COFs has also proven successful.^114−116^

While the intrinsic electrical conductivity of the COFs remains low, it can be augmented through chemical doping methods, thereby facilitating the formation of charge-transfer complexes. For instance, a fluorene-based 2D COF has demonstrated a high Seebeck coefficient of 2450 μV K^–1^ and a *PF* value of 0.063 μW m^–1^ K^–2^ at room temperature.^117^ Furthermore, a newly synthesized covalently bonded pyrene-based fully fused aromatic π-conjugated 2D organic network has exhibited an extraordinarily high hole mobility of 501 cm^2^ V^–1^ s^–1^ and a conductivity of 1038 S cm^–1^ at room temperature.^118^

**Figure 6:**
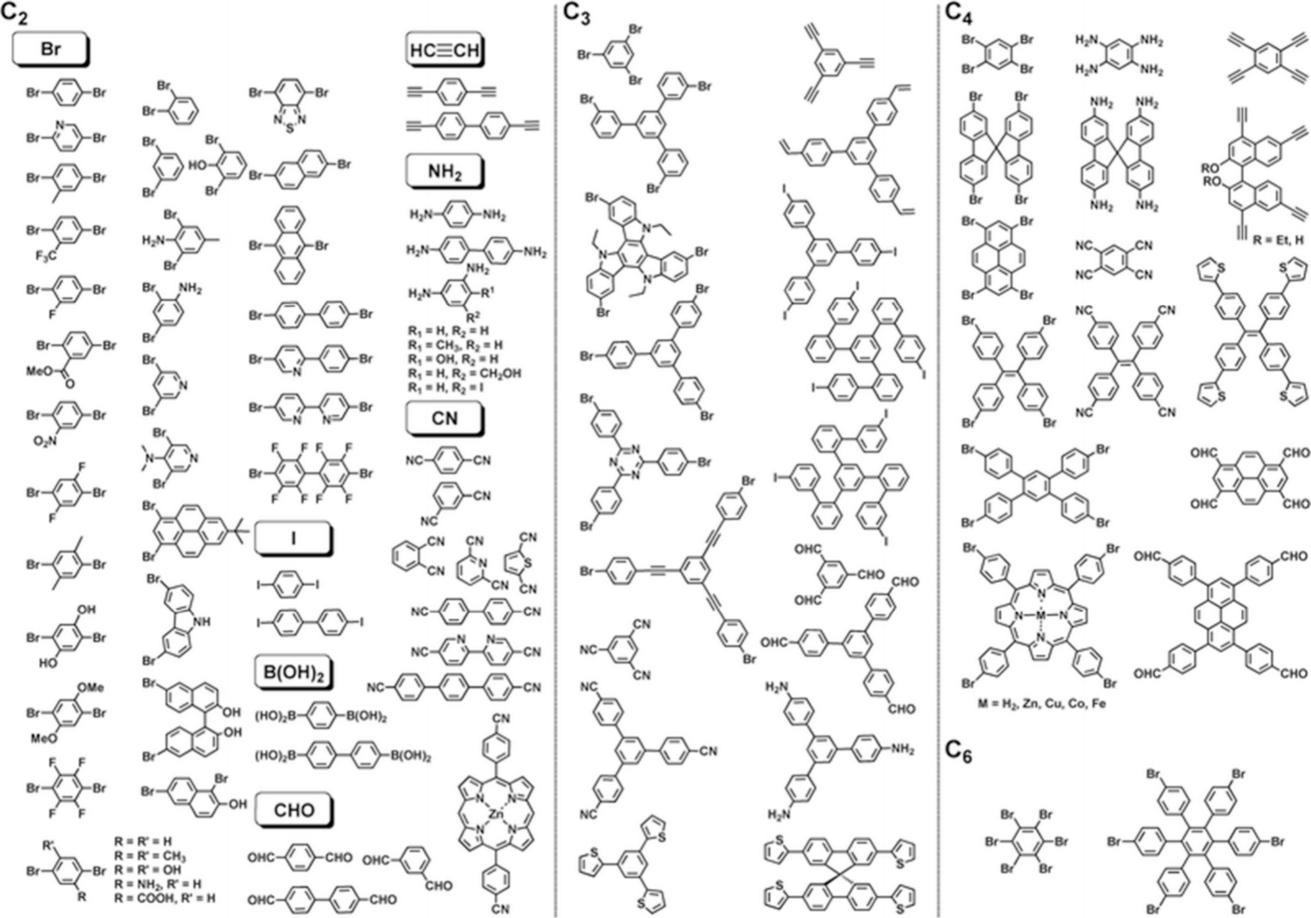
Representative building blocks with different geometries, sizes and reactive groups for the synthesis of CMPs. Reproduced from with permission from ref (121). Copyright 2013, Royal Society of Chemistry.

Alongside the COFs, other POPs also hold promise for thermoelectric applications. For instance, CMPs represent an advanced subclass of POPs characterized by their rigid π-conjugated structures, expansive specific surface areas, high thermal stability, and adjustable structures through synthetic diversification^119,120^ (Figure 6).^121^ The inherent porous nature of the CMPs renders them excellent candidates for achieving low thermal conductivity by facilitating efficient phonon scattering, thereby improving their thermoelectric performance for practical application. The unique combination of properties exhibited by CMPs, including their robust π-conjugated framework and tunable structure, positions them as promising candidates for further exploration and development in the field of thermoelectric materials.^122,123^

#### 3.1.3 Gels and Aerogels

**Figure 7:**
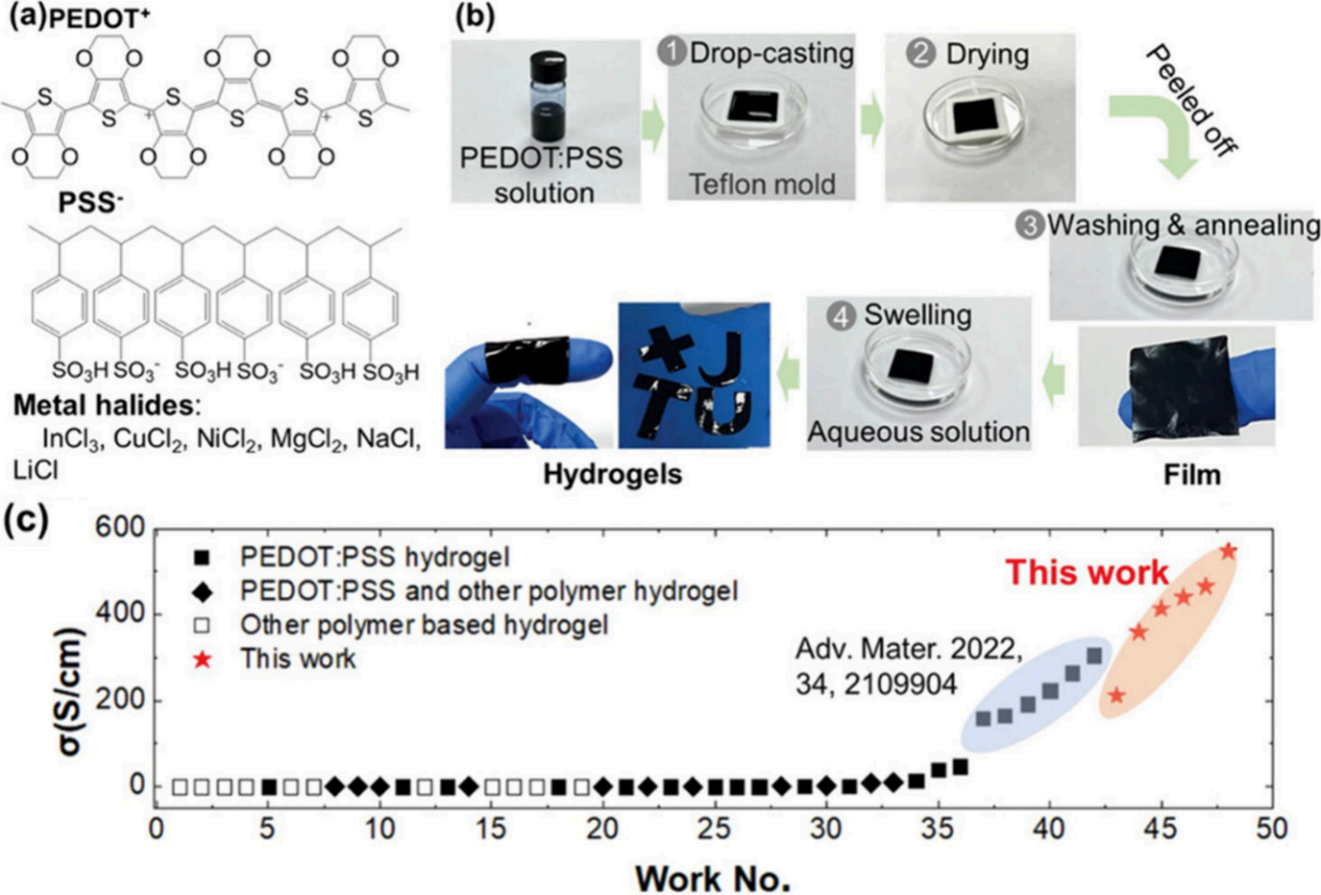
(a) Chemical structures of PEDOT+, PSS–, and metal halides. (b) PEDOT:PSS-based metal halide hydrogel preparation process with DMSO as the additive. (c) The comparison of the maximum electrical conductivity of PEDOT:PSS-MH hydrogels with polymeric hydrogels without conductive filler in the literature. Reproduced from with permission from ref (134). Copyright 2023, Wiley-VCH.

The gels and aerogels represent a particular class of meso- and macro-porous materials with low thermal conductivity due to their porous architectures.^124^ In addition, their structures offer practical versatility due to their favorable mechanical properties, such as flexibility, stretchability, and self-healing capabilities.^125,126^ The key challenge in realizing thermoelectric gels or aerogels lies in establishing a continuous conducting network for efficient carrier transport. Based on the type of connecting conductive network, gels can be categorized as either chemical gels or physical gels.^127,128^ Chemical gels achieve connectedness through covalent bonds, often via cross-linking or nonlinear polymerization processes. For instance, conductive gel precursors such as poly(3,4-ethylenedioxythiophene) (PEDOT), polypyrrole (PPy), or polyaniline (PANi) can undergo in situ polymerization via chemical oxidation.^129,130^ By contrast, physical gels rely on less energetic cooperative bonds such as hydrogen bonds or van der Waals interactions. The physical gel network forms through the aggregation of polymer chains due to phenomena such as hydrogen bonding, crystallization, complexation, or glassy junction points.^130^ These polymer chains create a framework within which the solvent is confined, with hydrogels being a prominent example in the case of water-based systems. Furthermore, various dried gels can be obtained by removing the solvent through different drying methods. Depending on the drying technique employed, dried polymer gels are classified as xerogels (ambient air drying), aerogels (supercritical drying), or cryogels (freeze-drying).^131,132^ Among these dried gel variants, aerogels are particularly advantageous for thermoelectric applications due to their unique supercritical drying process, which involves removal of the solvent by sequential freezing and sublimation. This process enables the preservation of the porous characteristics of the polymer network by avoiding collapse due to changes in the capillary stress via direct liquid-to-gas transition during drying.^132^ Consequently, the well-maintained porous structures of aerogels contribute to their lower thermal conductivity, thereby enhancing their suitability for thermoelectric applications. As an example, poly(3,4-ethylenedioxythiophene) polystyrenesulfonate (PEDOT:PSS) stands out as one of the most widely utilized conducting polymers, and is well suited for the formation of thermoelectric gels. It boasts several advantageous properties, including water solubility, ease of processing, mechanical flexibility, and relatively high electrical conductivity.^133^ For example, Wang et al. presented a groundbreaking study on metal-halide-doped PEDOT:PSS hydrogels with exceptional electrical conductivities of up to 547 S cm^–1^, exceeding those of previously reported filler-free polymeric hydrogels by as much as 1.5 to 10^4^ times (Figure 7).^134^ Theoretical calculations indicate that ion exchange between PEDOT:PSS and a metal halide plays a pivotal role in promoting phase separation within the hydrogels, thereby providing ultrahigh electrical conductivity. This, in turn, generates multifunctional properties, particularly in thermoelectric applications. The combination of flexibility, stretchability, and ultrahigh electrical conductivity, coupled with stability under various deformations, positions these hydrogels as promising candidates for wearable thermoelectric electronics. Other thermoelectric applications based on gels, such as thermogalvanic cells and ionic thermally chargeable capacitors, have been carried out in recent years. The porous polymer network not only provides the channels of ionic transport but also offers a large surface area, which enhances heat localization at the evaporation surface and minimizes heat loss and dissipation, therefore lowering thermal conductivity.^135^

**Figure 8:**
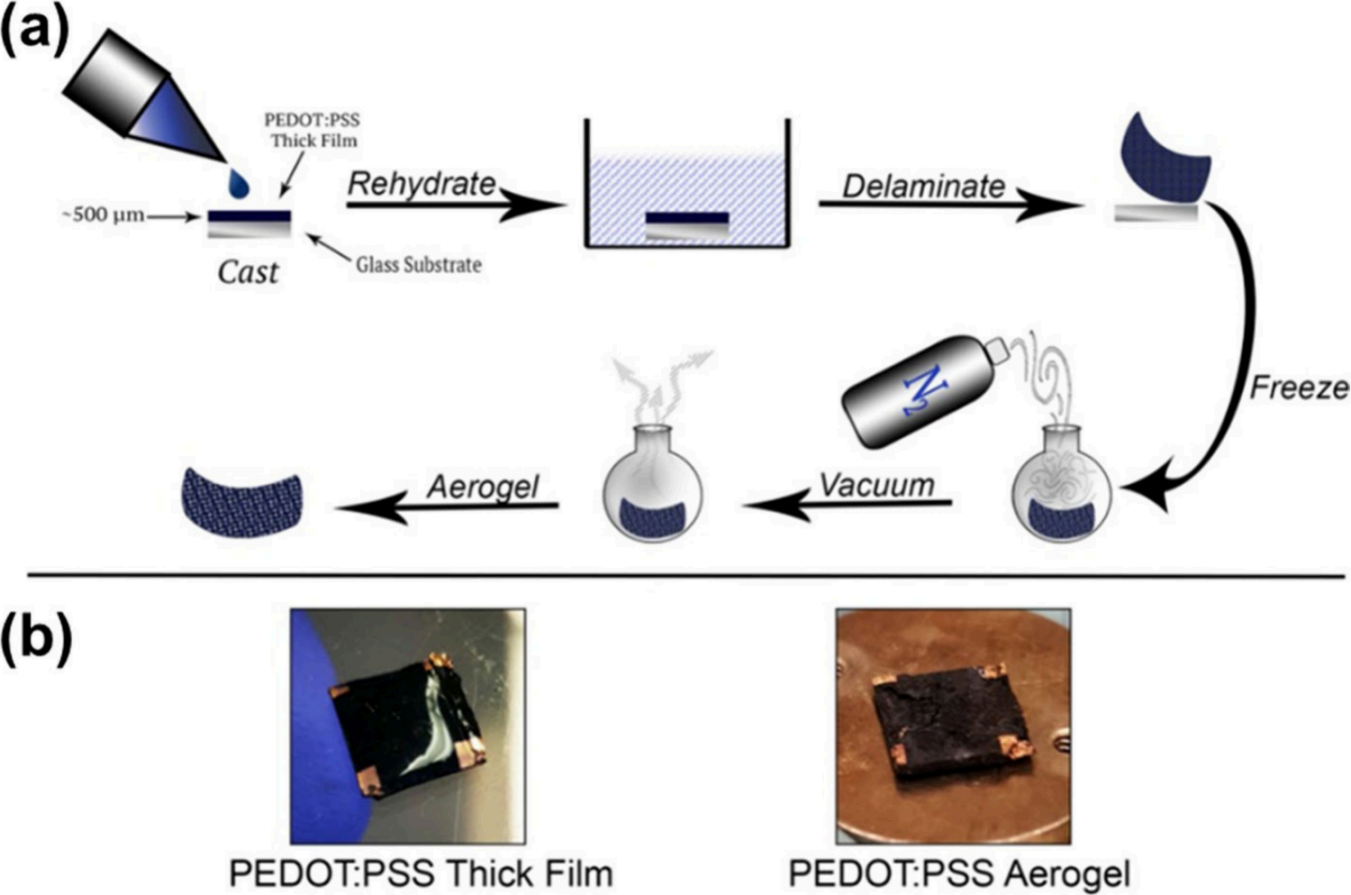
(a) Schematic illustrates the fabrication of PEDOT:PSS thick films and aerogels. (b) Photograph of macroscale morphology of both thick film and aerogel. Reproduced from with permission from ref (136). Copyright 2017, Wiley-VCH.

For dried polymeric gels, Gordon et al. proposed the utilization of ionic cross-linking to enhance the control over the network structure and elasticity of PEDOT:PSS aerogels.^136^ In their methodology, free-standing thick films of PEDOT:PSS are initially rehydrated to generate hydrogels, which are then subjected to freezing in liquid nitrogen and vacuum-pumped overnight (Figure 8).^136^ This procedure yields ultralight (density = 0.21–0.25 mg cm^–3^), robust, flexible, and macroporous PEDOT:PSS samples. The authors attribute the formation of the three-dimensional (3D) structure to the ionically cross-linked polymeric hydrogel network formed during the rehydration of the thick PEDOT:PSS film. The method yields a bulk porous structure with pore sizes ranging from 50 to 100 μm. However, these porous samples exhibit limited charge conductivity, typically in the range of a few S cm^–1^. To enhance their performance, the samples are soaked in ethylene glycol for various durations (2–30 min) to achieve a morphological transformation characterized by thicker walls and a more layered structure. This leads to an enhanced conductivity of ∼70 S cm^–1^ and a *PF* value of 1.8 μW m^–1^ K^–2^ for samples with a thickness of 500 μm, despite a slightly lower Seebeck coefficient of 16 μV K^–1^ on average.

### 3.2 High Electrically Conducting Fillers

The incorporation of low thermal conductivity materials into thermoelectric systems has been demonstrated to enhance the *zT* value by reducing the thermal conductivity. However, a prevailing challenge with such materials lies in their low electrical conductivity. Therefore, the inclusion of high electrically conducting fillers into these systems becomes crucial for achieving high *zT* thermoelectric composites. Representative high electrically conducting fillers in thermoelectric applications include conducting polymers, carbon nanomaterials, and various inorganic nanomaterials. The efficacy of incorporation of several high conducting filler is listed in Table 2. These are examined in more detail in the following subsections.

**Table 2:** Electrical Conductivity of Several Electrically Conducting Filler

Compound	Application	σ [S cm^–1^]	Reference
PEDOT:PSS-IL-WPU	thermoelectricity	∼140	(137)
PEO/F4TCNQ/P3HT	thermoelectricity	0.3	(138)
PDVT-10/SEBS/FeCl_3_	thermoelectricity	323	(139)
PEDOT:PSS/d-sorbitol	N/A	∼1000	(140)
PEDOT:PSS/xylitol	soft actuator	407	(141)
PEDOT:PSS/PEO20–PPO70–PEO20 (P123)	stretchable electrode	∼1700	(142)
SWCNT–TPU	thermoelectricity	∼100	(143)
PC/CNT/PC	thermoelectricity	∼289	(144)
CNT/PVAc	thermoelectricity	∼48	(145)
CNT/C-Dps	thermoelectricity	∼70	(146)
PγCyD/CNT	thermoelectricity	∼3000	(147)
PVAc/G/INC	thermoelectricity	218	(148)
PVC/n-PETT/CNT	thermoelectricity	630	(149)
CNT/graphite/poly(lactic acid)	thermoelectricity	∼41	(150)
PVP/Ag/Ag_2_Te	thermoelectricity	361	(151)
Ag_2_Se/resin	thermoelectricity	∼100	(152)
PDI-Te	thermoelectricity	∼0.9	(153)
Ni NW/PVDF	thermoelectricity	4701	(154)
Ag_2_Se NW/PVDF	thermoelectricity	206	(155)
Ni NW/PVDF	thermoelectricity	253	(156)
Cu_2__–_*_x_*Se (*x* ≥ 0.25) NW/PVDF	thermoelectricity	∼5100	(157)

#### 3.2.1 Conducting Polymers

The discovery of high conductivity in I_2_-doped polyacetylene (PA) marked a significant milestone in the realm of conducting polymers, paving the way for the design and synthesis of conducting polymers such as PPy, polythiophene (PTh), PANi, and PEDOT and catalyzing advancements in organic electronics.^158,159^ These polymers exhibit semiconducting properties due to the presence of delocalized π-bonds along their conjugated backbones, coupled with relatively low thermal conductivity compared to inorganic compounds, which makes them potential candidates for thermoelectric materials.^137,160^ However, the first generation of conjugated polymers faced challenges such as insolubility and infusibility, which limited their utility in practical applications. Therefore, the development of solution-processable conjugated polymers became paramount for the fabrication of large-scale thermoelectric devices. For instance, PEDOT:PSS is a polyelectrolyte comprised of positively charged electrically conducting conjugated PEDOT and negatively charged insulating PSS. The insoluble PEDOT short chains adhere to the water-soluble PSS long molecular chains via Coulombic forces, thereby forming a stable dispersion in water.^161^ This method stands out as a significant technical breakthrough in enhancing the processability of intrinsically conductive polymers.

Due to its numerous advantages, including easy doping tunability, high transparency, mechanical flexibility, thermal stability, and solution-processability, PEDOT:PSS has emerged as the most successful conducting polymer in various applications.^162^ Leveraging its excellent solution-processability, PEDOT:PSS can be blended with other polymers to create organic thermoelectric composites. For example, Taroni et al. blended commercial elastomeric polyurethane (Lycra^Ⓡ^) with PEDOT:PSS in dimethyl sulfoxide (DMSO) to develop stretchable thermoelectric systems suitable for wearable applications.^163^ The phase structure morphology of the PEDOT:PSS and Lycra blends was meticulously characterized, revealing Lycra dispersed as fibers with diameters of approximately 100 μm in the PEDOT:PSS matrix. Notably, a 10 wt% PEDOT:PSS-Lycra film achieved an unprecedented strain at breaking point of 700%. Moreover, post-treatment with ethylene glycol yielded a high electrical conductivity of 79 S cm^–1^ and a Seebeck coefficient of 16 μV K^–1^. Consequently, the blend was used to fabricate a self-powered sensor that harnessed the thermoelectric effect of PEDOT:PSS to enable the composite films to detect tensile stress without the need for an external power supply. Moreover, PEDOT:PSS can also be effectively combined with other conjugated polymers to create a significant energy filtering effect. For instance, Jo et al. demonstrated a PEDOT:PSS/PANi thermoelectric composite with innovative multilayered structures.^164^ As the number of repeated deposition cycles increased, the electrical conductivity of the multilayer composite films correspondingly increased. Specifically, with 4 deposition cycles, the electrical conductivity and *PF* value reached 1550 S cm^–1^ and 56 μW m^–1^ K^–2^, respectively, surpassing those of the neat PEDOT:PSS by 1.3 and 2 times. The multilayer structure induced stretching of the PEDOT and PANi chains within the composite, which facilitated effective hole diffusion between the PANi and PEDOT:PSS layers, thereby enhancing the electrical conductivity and thermoelectric performance.

PEDOT:PSS can also be combined with porous materials to form high electrical conductivity and low thermal conductivity thermoelectric nanocomposites. For example, Deng et al. demonstrated flexible and foldable thermoelectric devices based on the Chinese traditional Xuan paper filled with PEDOT:PSS polymers.^165^ By a dip-coating and subsequent drying process, the nanocomposites film reached a high electrical conductivity of about 2213 S cm^–1^, which resulted from the vacuum process and the high hydrophilicity and porous 3D network structure, making the PEDOT:PSS polymers efficiently filled with Raw Xuan paper.

#### 3.2.2 Carbon Nanomaterials

**Figure 9:**
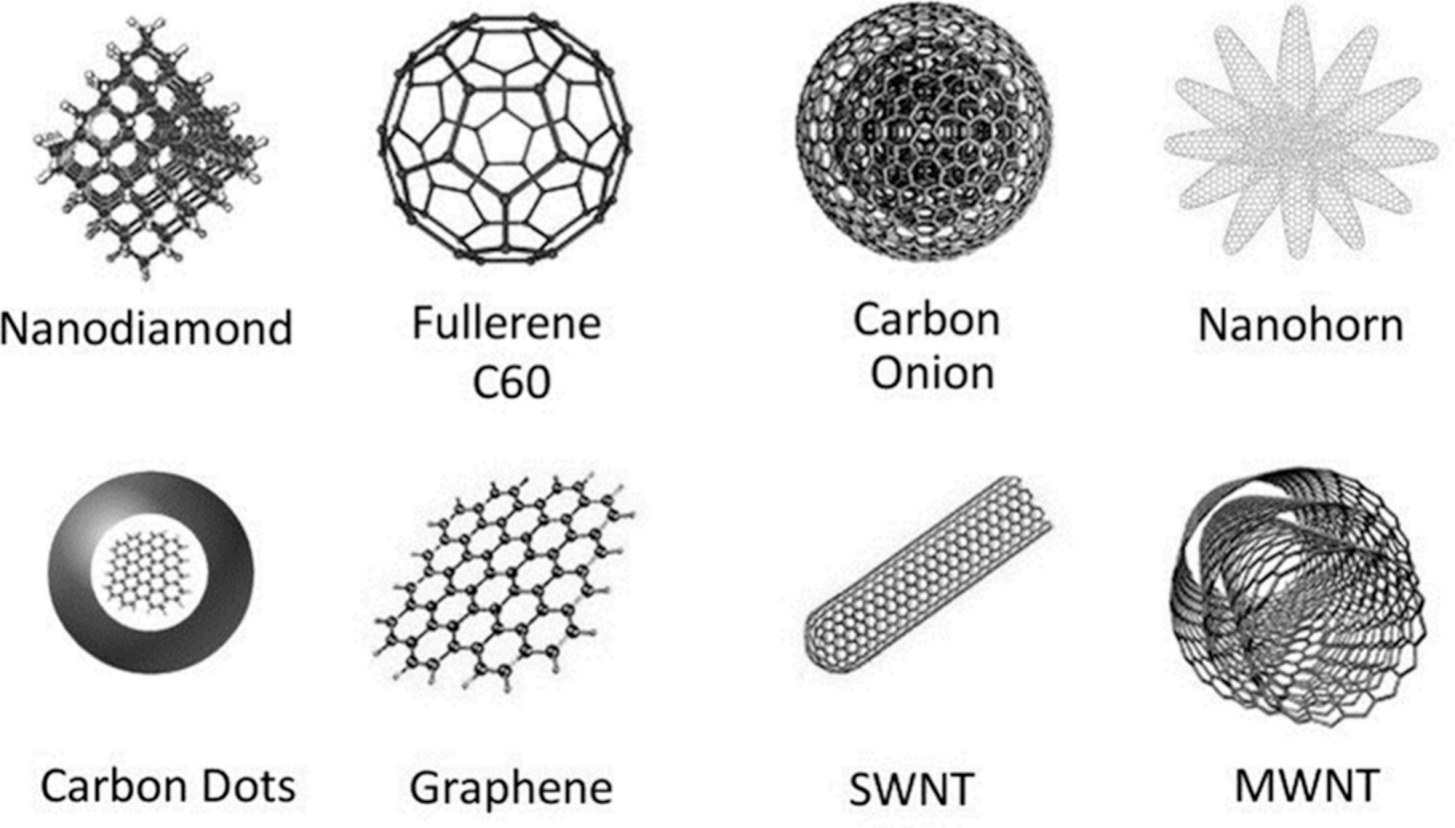
Typical carbon nanomaterial family. Reproduced from with permission from ref (166). Copyright 2015, Royal Society of Chemistry.

Approximately a decade ago, researchers began exploring the utilization of these unique nanoscale carbon systems in thermoelectric composites due to their remarkable characteristics, including high electrical conductivity, large specific surface area (leading to enhanced interfacial interactions), flexibility, low weight, and high mechanical strength (Figure 9).^47,166^ Composites comprised of CNTs and graphene with polymers or inorganic materials hold the potential to decouple the electrical and thermal conductivity by introducing interfaces that offer more efficient pathways for electron transfer compared to phonon transfer. Furthermore, the introduction of interfaces also creates opportunities for carrier energy filtering, thereby decoupling the Seebeck coefficient and electrical conductivity.^50,167^ This innovative approach enables the optimization of thermoelectric performance by tailoring the material’s electronic properties while simultaneously mitigating thermal transport. For instance, various insulating polymers such as gum arabic and polyvinyl acetate have been utilized to fabricate composites with CNTs. These composites leverage the insulating polymer as a binder to stabilize an interconnected CNT network. For example, Mo et al. developed cellulose acetate/single-walled carbon nanotube (SWCNT) composites using a bar-coating method.^168^ These composites exhibited a remarkable *PF* value of 140 μW m^–1^ K^–2^ for p-type transport and could easily be subjected to n-type doping by painting the freestanding films with a polyethylenimine (PEI) solution. The exceptional performance was attributed to the presence of secondary aggregates, namely bundles of bundles, which reduced the interbundle resistance. More recently, Hata et al. demonstrated composites consisting of CNTs embedded in a γ-cyclodextrin polymer cross-linked with epichlorohydrin (PγCyD).^169^ These composites exhibited *PF* values exceeding 200 μW m^–1^ K^–2^ for both p-type and n-type transport. The variation in majority carrier type was attributed to distinct interactions with the processing solvent–either H_2_O or *N*-methyl-2-pyrrolidone (NMP). Specifically, a “pocket” in the γCyD macrocycle can host the NMP molecule, thereby enhancing the n-type behavior.

Various nanocarbon fillers, including graphene, have also been explored for composite materials. For instance, the noncovalent attachment of a heavily fluorinated C_60_ fullerene to the surface of reduced graphene oxide (rGO) incorporated into PEDOT:PSS has resulted in an enhanced *PF* value compared to both pristine PEDOT:PSS and PEDOT:PSS-rGO composite samples.^170^ This is primarily attributed to an improved Seebeck coefficient from the filtering of low-energy carriers at the interface between the PEDOT:PSS and the rGO-C_60_ fullerene fillers, facilitated by the Schottky barrier.

Other systems of conducting polymer-based carbon composites, such as PEDOT/CNT, PANi/CNT, PPy/CNT, PEDOT/graphene, PANi/graphene, PPy/graphene, and ternary-component systems are recommended to refer to the review paper by Zhang et al.^171^

#### 3.2.3 Metallic and Semiconducting Materials

**Figure 10:**
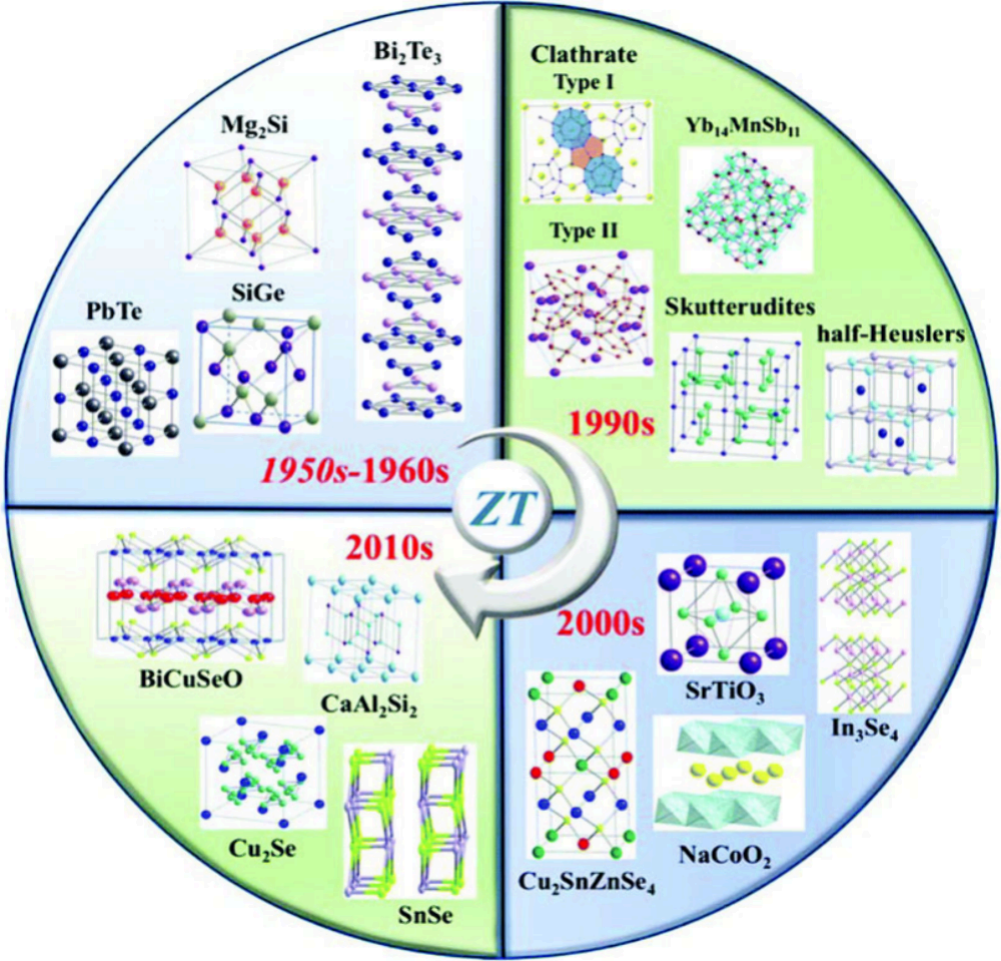
Representative inorganic thermoelectric materials from the 1960s to the present. Reproduced from with permission from ref (10). Copyright 2018, Royal Society of Chemistry.

Aside from high conducting carbon-based nanomaterials, the incorporation of nanostructured inorganic materials into polymer composites is a promising strategy for enhancing the performance of thermoelectric materials.^155,172^ Nanostructured inorganics such as Te, Bi_2_Te_3_, tin selenide (SnSe), molybdenum disulfide (MoS_2_), etc., offer high electrical conductivity and Seebeck coefficients, which can be coupled effectively with the low thermal conductivity of polymers (Figure 10).^10^ This combination is advantageous for achieving high thermoelectric efficiency.

The main benefit of using polymer composites with nanostructured inorganics is the ability to tune the electrical conductivity independently from the Seebeck coefficient, while benefiting from the low thermal conductivity of the polymer matrix. This approach allows for tailoring of the thermoelectric properties to optimize the *zT* of the composite material. For example, materials such as Bi_2_Te_3_ and SnSe are well-known for their high Seebeck coefficients, making them ideal candidates for enhancing the thermoelectric properties of composites. When combined with polymers, these materials can potentially achieve a good balance between electrical conductivity and Seebeck coefficient, thus leading to an enhanced thermoelectric performance. For instance, Zaia et al. coated Te nanowires with perylene diimide (PDI) to achieve a stable organic composite known as PDI-Te.^153^ Various volume ratios of PDI were evaluated during synthesis, with the highest *PF* value of 17.6 μW m^–1^ K^–2^ being achieved at 80 vol % PDI. Meanwhile, in a study focused on scalable production, Chen et al. embedded nickel (Ni) nanowires within a polyvinylidene difluoride (PVDF) matrix without doping.^154^ The researchers prepared n-type Ni/PVDF films by blending Ni nanowires with PVDF powder in dimethylformamide (DMF) solution. The results demonstrated that increasing the Ni nanowire content from 20 to 80 wt% enhanced the carrier mobility and significantly improved the electrical conductivity. The study concluded that the n-type Ni/PVDF films achieved a notably high *PF* value of 200 μW m^–1^ K^–2^ with 80 wt% Ni nanowires.

Inorganic compounds can also be blended with conjugated polymer systems to enhance their electrical characteristics. For instance, Ju et al. prepared a hybrid thermoelectric composite by incorporating SnSe nanosheets into a conducting PEDOT:PSS polymer matrix.^173^ The results showed that while the addition of SnSe nanosheets increased the Seebeck coefficient of the composites, it reduced the electrical conductivity. Nevertheless, an optimal content of 20 wt% SnSe nanosheets resulted in the highest *PF* value of 390 μW m^–1^ K^–2^ at room temperature.

In addition to being blended with organic compounds, the inorganic thermoelectric compounds could also combine with other porous materials. For instance, Zhang et al. developed a novel mid-temperature thermoelectric material based on Bi_0.4_Sb_1.6_Te_3_ combined with MOFs featuring 1-by-1 nm pore engineering.^174^ Uniform dispersion of four types of MOFs (ZIF-8, UiO-66, MIL-101, and MOF-919-Fe) within the Bi_0.4_Sb_1.6_Te_3_ matrix was achieved using spark plasma sintering (SPS), preserving their microstructural integrity. This integration resulted in enhanced thermoelectric performance through efficient electron transport at the organic–inorganic interfaces and reduced thermal conductivity due to strong phonon scattering within the nanoporous framework. The maximum *zT* of 1.65 was obtained for Bi_0.4_Sb_1.6_Te_3_/0.5 wt% ZIF-8 at 348 K.

## 4 Summary and Outlook

This review has examined two classes of materials used for thermoelectric composite systems, each demonstrating the potential to enhance the overall thermoelectric performance through distinct advantages. For instance, incorporating metal–organic frameworks (MOFs) can significantly lower the thermal conductivity and enhance the figure of merit (*zT*). However, several areas still require improvement for future thermoelectric applications.

From a performance perspective, the current materials do not yet meet the practical usage requirements. For example, a *zT* value greater than 3 is required for efficient solid-state cooling to replace freon-based conventional refrigerators, while a *zT* greater than 5 is required in order to attain twice the efficiency of traditional combustion engines. Therefore, the continued pursuit of materials with high *zT* values is crucial for further commercialization. To achieve high thermoelectric performance, it is essential to develop design rules that effectively analyze the structure–property correlations of various promising organic thermoelectric material systems. For example, in the study of low thermal conductivity materials, the precise relationship between structure and thermoelectric properties needs to be elucidated. Specifically, in the design of MOFs for thermoelectric applications, the impacts of the chemical structure and pore geometry on the thermoelectric performance have thus far been discussed primarily through computational studies. At the molecular scale, specific chemical structures inherently dictate the corresponding pore architectures, as exemplified by MOFs and COFs. However, challenges persist in controlling reaction purity and crystallization during synthesis. One potential strategy to address these challenges is the employment of advanced techniques such as molecular layer deposition (MLD) or atomic layer deposition (ALD) for MOF synthesis. These layer-by-layer epitaxy methods can enhance control over MOF growth and the resultant pore structures. On a macroscopic scale, freeze-drying is a commonly utilized method in aerogel fabrication that helps maintain pore structures. Adjusting the freeze-drying parameters can influence pore size and distribution. Additionally, specialized pore control methods, such as the shearing method,^175^ may be referenced in future work focused on the development of thin-film porous thermoelectric devices.

The other factor to influence a *zT* value is a *PF* value. A low *PF* value in organic porous materials primarily stems from their poor electrical conductivity. Therefore, increasing electrical conductivity is the most straightforward approach to enhancing the *PF* value. This can be achieved through various strategies, such as introducing conducting fillers, introducing guest molecules,^176^ optimizing material design, or modifying the postsynthetic form of the MOFs. For instance, if the synthesized material is an insoluble powder, it is usually processed by pellet-press method, which can lead to porosity issues and reduced electrical conductivity. The same MOF in different forms (e.g., thin films vs powders) can exhibit distinct electrical conductivity levels. Hence, improving the synthesis methods is another potential solution. An example is Ni_3_(HITP)_2_, which can be fabricated as thin films, electrochemical assemblies, or powders.^60^ Exploring such versatile processing methods can help overcome conductivity limitations.

In addition, at the microscopic level, charge carrier transport in porous organic materials, such as MOFs, COFs, and CMPs, is predominantly influenced by their chemical structure and crystallinity, similar to polymer-based thermoelectrics. The presence of ordered π–π stacking and strong electronic coupling between adjacent units facilitates band-like transport. In contrast, defects or disordered regions typically lead to hopping transport. Therefore, optimizing the chemical structure to enhance crystallinity and π-conjugation is essential for improving charge mobility.

At the macroscopic level, the morphology and connectivity of the porous structure significantly influence the overall charge transport pathways. The inherent porosity of these materials poses challenges in forming a continuous conductive network. For example, in aerogels or porous films, the connectivity between different regions of the framework is crucial for establishing a percolation network, which greatly impacts electrical conductivity. If this network is not well-formed, charge carriers may become trapped or localized, resulting in diminished thermoelectric performance.

Additionally, Practical experiments and systematic discussions are still lacking, and are needed in order to build a comprehensive design guideline. An understanding of the interactions between low thermal conductivity materials and high electrical conductivity fillers is also crucial. These interactions play significant roles in optimizing the overall performance of the thermoelectric composites. Hence, future research should also focus on both computational findings and experimental validation of these interactions to pave the way for high-performance thermoelectric composite systems. The introduction of guest molecules through doping strategies represents a promising avenue for enhancing the electrical conductivity of OPMs. Current approaches predominantly utilize sequential doping techniques, wherein dopants are incorporated postsynthetically. However, the selection of dopants remains constrained, highlighting the need to expand the library of potential dopants to optimize the electronic properties of these materials. Future research endeavors should prioritize the exploration of possible dopants, including small organic molecules, metal ions, and redox-active species, which can interact with the material framework without compromising its structural integrity. Ideal dopants would not only modulate charge carrier concentration and mobility but also maintain a high Seebeck coefficient. Additionally, efforts should be directed toward fine-tuning doping levels and developing more efficient doping methodologies that leverage the inherent porosity and tunable nature of these frameworks to maximize thermoelectric performance.

Anisotropic properties of electrical and thermal conductivity are another issue to influence the thermoelectric performance. While MOF-organic hybrid thermoelectric materials have shown great potential, one critical aspect that requires further investigation is their anisotropic thermoelectric behavior. Currently, there is a noticeable gap in the literature regarding the anisotropic properties of these hybrid systems. However, studies on individual components, such as MOFs and organic thermoelectrics, suggest that their hybrids are also likely to exhibit significant anisotropic electrical and thermal conductivities. For instance, anisotropy in electrical conductivity has been demonstrated in specific MOF systems due to directional charge transport pathways influenced by the coordination network structure.^177,178^ Similarly, pure organic thermoelectric materials, such as PEDOT, have shown that molecular alignment and the nature of the π-conjugated backbone can result in anisotropic transport behavior.^179^ In light of these observations, it is essential to develop experimental and theoretical approaches to systematically explore the anisotropy in MOF-organic hybrid materials. Such insights would not only refine the understanding of their fundamental transport mechanisms but also aid in optimizing their thermoelectric performance through strategic material design. Future research in this direction could significantly enhance the applicability of these hybrid materials in practical thermoelectric devices, where directional thermal and electrical conductivities could be leveraged for improved efficiency and functionality.

The other challenge is the stability of porous thermoelectric materials. Some methods to enhance both the chemical and thermal stability of MOFs have been discussed, such as strengthening the coordination bond by choosing high-valent metals, choosing hydrophobic ligands, and choosing a metal ion at its most stable oxidation state.^180^ Besides, in 2022, Escobar-Hernandez et al. presented an advanced predictive model for determining the thermal stability of MOFs by computational chemistry and machine learning algorithm.^181^ Future designs could be considered to create wearable thermoelectric devices.

With respect to practical application, numerous foreseeable challenges still limit the development of these materials. For example, the limited processability of these novel materials poses significant hurdles. To realize large-scale applications, fabrication methods need substantial improvement. For composite materials, achieving scalability often requires enhancing interfacial interactions or employing in situ polymerization techniques, both of which are conducive to mass production. For example, in situ polymerization has been successfully utilized in the synthesis of conjugated microporous polymer–graphene composite films, demonstrating great promise for high-efficiency, flexible devices.^19,182^ Regarding pure porous materials, approaches such as layer-by-layer epitaxy are particularly well-suited for scalable production.^183^ This technique allows for the fabrication of uniform and well-defined thin films, providing high programmability and enabling the creation of high-quality, reproducible MOFs. Additionally, interface reaction-based synthesis represents another effective approach, as it facilitates the production of large-area materials if the reaction vessel is adequately scaled up.^184^ However, the collateral impacts of these improvements must be carefully considered and managed within the complex composite systems. By addressing these challenges and refining the fabrication processes, it is believed that practical high thermoelectric devices can be achieved. This advancement would significantly promote the practical applications of renewable and continuous heat-electricity conversion technologies.
